# Polymeric Desensitizer Fluororubber: A Good Binder to Improve the Thermal Stability and Mechanical Properties of 3,4-Dinitrofurazanfuroxan

**DOI:** 10.3390/molecules30081665

**Published:** 2025-04-08

**Authors:** Shenghui Wang, Xiaogang Mu, Yiming Luo, Ronghui Ju, Xuanjun Wang, Haixia Ma, Jijun Xiao

**Affiliations:** 1 Zhijian Laboratory, Rocket Force University of Engineering, Xi’an 710025, China; wshalano@163.com; 2 Xi’an Modern Chemistry Research Institute, Xi’an 710065, China; iamrlym@126.com (Y.L.); ronghuiju@126.com (R.J.); 3 Xi’an Key Laboratory of Special Energetic Materials, School of Chemical Engineering, Northwest University, Xi’an 710069, China; mahx@nwu.edu.cn; 4 Molecules and Materials Computation Institute, School of Chemical Engineering, Nanjing University of Science and Technology, Nanjing 210094, China

**Keywords:** 3,4-dinitrofurazanfuroxan, fluororubber, molecular dynamics simulation, differential scanning calorimetry, thermal stability, mechanical properties

## Abstract

3,4-Dinitrofurazanfuroxan (DNTF) is characterized by its high energy, high detonation velocity, strong explosive power, and small critical diameter for detonation. However, its practical application is limited by poor thermal stability and mechanical properties. In this study, the polymeric desensitizer fluororubber (F2603) was introduced as a binder to enhance the overall performance of DNTF. Molecular dynamics (MD) simulations were used to investigate the thermal stability (trigger bond length and cohesive energy density (CED)) and mechanical properties, including elastic coefficient (*C*_ij_), tensile modulus (*E*), bulk modulus (*K*), shear modulus (*G*), Cauchy pressure (*C*_12_*–C*_44_), and Poisson’s ratio, for both pure DNTF (1 1 1) and DNTF (1 1 1)/F2603 composite systems at varying temperatures. The thermal stability was further experimentally investigated using differential scanning calorimetry (DSC) technique. The results demonstrated that the addition of F2603 leads to a shorter trigger bond length, higher CED, and a 7.2 kJ·mol^−1^ increase in activation energy (Ea), indicating improved thermal stability. Additionally, mechanical property simulations indicated that F2603 decreased the *E*, *K*, and *G* of DNTF while increasing the K/G ratio, suggesting enhanced mechanical toughness. These studies have important implications for the formulation design and practical application of DNTF and its composites.

## 1. Introduction

**Figure 1:**
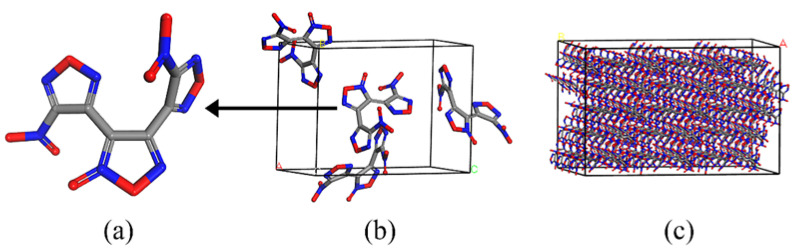
The molecular structure of DNTF (**a**); single crystal (**b**); 4 × 3 × 5 supercell (**c**).

Polymer bonded explosives (PBXs) are composite explosives that offer excellent detonation properties, high energy, superior formability, and processability. These characteristics make them crucial in military, aerospace, and other fields, as they consistently demonstrate strong safety performance, mechanical strength, and resistance to storage degradation. Generally speaking, this kind of explosive is composed of a main explosive, a polymer passivant, and many other additives [1,2,3,4], of which the most important components are the main explosive and the polymer passivant. The commonly used explosives are trinitrotoluene (TNT) [5], cyclotrimethylnitrosamines (RDX) [6], cyclotetramethylenetetramine (HMX) [7], hexanitrohexaazaisowurtzitane (CL-20) [8], 3,4-dinitrofurazanfuroxan (DNTF) [9], etc. Among the above explosives, DNTF stands out with superior energy, density, detonation velocity, power, detonation critical value, and casting performance compared to TNT, RDX, HMX, and CL-20, leading to increased research interest in recent years [10,11]. The molecule structure, single crystal, and (4 × 3 × 5) supercell structures of DNTF are shown in Figure 1. However, due to its high sensitivity, DNTF can compromise the safety of molten-cast explosives, limiting its practical applications [12]. To mitigate this issue, the addition of a polymer passivator to the DNTF is considered to be an effective approach [13].

Numerous foundational studies have focused on high-energy polymer explosives, examining various experimental aspects such as spectroscopy, energy spectra, thermal analysis, and sensitivity testing [14,15,16]. However, selecting polymer materials that effectively interact with the primary explosive often involves significant experimental costs [17]. Furthermore, explosives have their own oxidation and decomposition characteristics, that temperature exceeding some certain degree will accelerate the reaction speed of decomposition. This can lead to deflagration or explosion, posing potential safety risks. For this reason, people have been eager to strengthen the basic theoretical research of formulation design. There are not many theoretical studies on the thermal stability and mechanical properties of DNTF with polymer insensitive agents. Molecular dynamics (MD) simulations have been employed in the study of energetic explosives and the simulation results proved to be in good agreement with the experimental data [18,19,20].

In this paper, the fluoropolymer F2603, which has excellent heat resistance and physical and mechanical properties, is selected as the polymeric passivator [21] to build a mixed explosive model, and the MD simulation of the DNTF (1 1 1) system and the DNTF (1 1 1)/F2603 composite system is carried out. The thermal stability (trigger bond length, cohesive energy density) and mechanical properties (elastic coefficient (*C*_ij_), tensile modulus (*E*), bulk modulus (*K*), shear modulus (*G*), and cauchy pressure (*C*_12_–*C*_44_), *K*/*G*,) of both systems were predicted at various temperatures. Subsequently, the thermal stability and mechanical properties of the DNTF (1 1 1) system and the DNTF (1 1 1)/F2603 composite system as a function of temperature were analyzed. Additionally, the thermal decomposition characteristics of the two systems were analyzed using differential scanning calorimetry (DSC) techniques. The investigation provides a theoretical reference for the formulation design and practical application of DNTF–based hybrid explosives.

## 2. Simulation Details

### 2.1. Model Construction

The COMPASS force field, a powerful all-atomic force field, was employed in this study to model the structure and properties of both individual and condensed materials [22,23,24,25,26]. It uses a complex set of functions to accurately describe the interactions between substances. The nitro groups have been explicitly parameterized and included in the COMPASS force field, which has good applicability to high-energy materials. Previous studies on DNTF have confirmed that the COMPASS force field is suitable for high-energy mixed explosive systems [27,28]. Therefore, the whole work in this paper is carried out under the COMPASS force field.

Generally, the mole fraction of F2603 in DNTF is 2.4% [29]. A single F2603 polymer chain and its 3D model was constructed using the Amorphous Cell module in Materials Studio software (Version 8.0). The optimization algorithm employed was Smart, with the precision set to Fine, and the maximum number of iterations was set to 50,000 steps. The allowed deviations for the energy, force, stress, and displacement were set to 0.0001 Kcal·mol^−1^, 0.05 Kcal·mol^−1^·Å^−1^, 0.005 GPa, and 0.00005 Å, respectively. To further relax the amorphous model of F2603, molecular dynamics (MD) simulations were performed under the NPT ensemble. The temperature was set to 298 K, and the COMPASS force field was used. The temperature was controlled using the Anderson thermostat [29], and pressure was maintained using the Berendsen barostat [30]. Atom-based [31] and Ewald [32] methods were used to calculate van der Waals force (vdW) and electrostatic force (Coulomb), respectively. The final structure obtained is the equilibrium configuration of polymer F2603.

**Figure 2:**
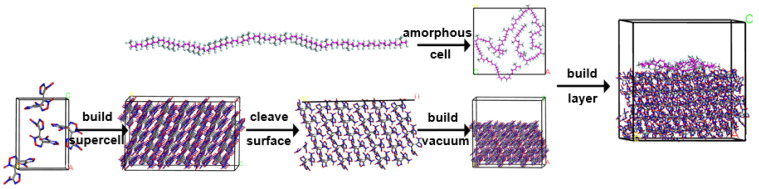
The procedure of model establishment of DNTF (1 1 1)/F2603.

The DNTF crystal structure was obtained based on the Cambridge database (CCDC 270417) and used as the initial structure for this paper. The cell parameters of DNTF are *a* = 6.662 Å, *b* = 10.740 Å, c = 15.093 Å, and *α* = *β* = *γ* = 90°, and the space group is P_2_, _2_, _2_. In order to verify its suitability for the built system, the structure of the DNTF cell was optimized by COMPASS force field. As shown in Table 1, the differences between the simulated and experimental cell parameters are minimal (within 5.0%), indicating that the COMPASS force field is appropriate for this system. Previous studies [33] have proved that the (1 1 1) surface is the largest and most important crystal surface of DNTF in crystal morphology, indicating that this crystal surface, as the boundary of DNTF crystal, has the greatest chance of contacting with polymer insensitive agent and has good comprehensive performance. Therefore, DNTF (1 1 1) surface was selected as the basic explosive system to carry out the whole work. First, the initial cell of DNTF was expanded to (4 × 3 × 5) supercell and cut along the direction of (1 1 1) crystal plane. Then, the amorphous units of polymer F2603 were added to the optimized (1 1 1) crystalline surface, constructing a DNTF (1 1 1)/F2603 composite system consisting of 5672 atoms. Finally, the DNTF (1 1 1)/F2603 composite system was continuously compressed along the c-axis, bringing it closer to the theoretical density of 1.93 g·cm^−3^. The whole procedure is illuminated in Figure 2.

### 2.2. MD Simulation

The optimized system is imported as the original file of the MD simulation in the Forcite module. Firstly, the DNTF (1 1 1)/F2603 composite system was simulated for 500 ps at 298 K with NVT and COMPASS force field to initially relax the hybrid system. To account for the temperature and computational resource constraints of both the DNTF (1 1 1) system and the DNTF (1 1 1)/F2603 composite system during application, five temperatures were selected under atmospheric pressure (0.1 MPa) using the NPT ensemble: 298 K, 323 K, 348 K, 373 K, and 398 K. These simulations aimed to study the system’s performance. The total simulation time was 1000 ps, with a time step of 1 fs.

### 2.3. Materials and DSC Testing

Materials: DNTF, purity >99%, provided by Xi’an Modern Chemistry Research Institute; F2603, Chenguang Chemical Factory. DNTF and F2603 were mechanically mixed with the ratio of 95:5. The thermal behavior of DNTF and DNTF/F2603 mixture was tested on a 200F3 differential scanning calorimeter (Netzsch, Selb, Germany). Samples weighing approximately 0.2 mg were heated at rates of 5, 10, 15, and 20 K·min^−1^. The experiments were conducted within a temperature range of 50 to 400 °C in the nitrogen atmosphere with a flow rate of 5 mL·min^−1^ in sealed crucibles.

## 3. Results and Discussion

### 3.1. Judgment of System Equilibrium and Equilibrium Structure

**Figure 3:**
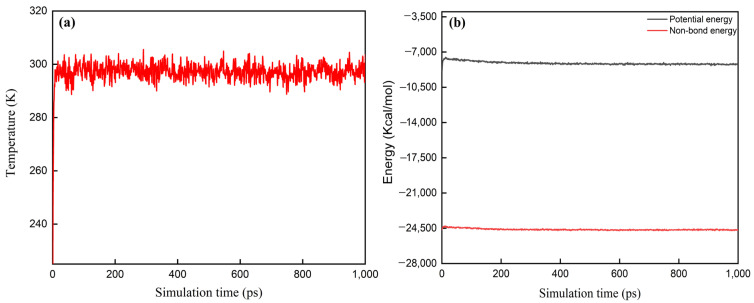
Temperature–time (**a**) and energy–time (**b**) equilibrium curves of the DNTF (1 1 1)/F2603 composite system at 298 K.

The transition between stable structures may involve energy barriers, which require the system to overcome a certain activation energy. These energy barriers can cause the system to become “trapped” in local minima (metastable states), necessitating longer simulation times or enhanced sampling techniques to explore transitions to other stable structures [34]. The objective of this study is not to track all possible metastable states, but rather to simulate the system once it reaches equilibrium and analyze the system’s thermal stability and mechanical property parameters through statistical analysis of the equilibrium trajectory. The calculation of these macroscopic properties relies on the average values of the equilibrium trajectory, rather than the dynamic processes of specific instantaneous conformations. Therefore, in thermodynamic systems, when temperature and energy fluctuations remain within the range of 5% to 10%, it indicates that the system has reached equilibrium [35]. Figure 3 shows the fluctuation curves of temperature and energy during the MD simulation of the DNTF/F2603 composite system. As can be seen from Figure 3, the temperature value of the system fluctuates around 298 K. The potential energy and non-bond energy of the system fluctuate around −8178 Kcal·mol^−1^ and −24,696 Kcal·mol^−1^, respectively, with fluctuations within 10%, indicating that the mixed system reached equilibrium. The other models are also at equilibrated state based on the criteria.

### 3.2. Thermal Stability

The thermal stability of energetic materials (EMs), defined as their susceptibility to accidental combustion or explosion under external heating, is a critical safety concern. Researchers have proposed many methods on how to estimate the thermal stability of Ems [36,37]. In this paper, we use a new theory proposed by Xiao Jijun et al. [38,39] to judge the thermal stability of DNTF and its composite system. They pointed out that the length of the trigger bond and cohesive energy density are direct indicators of thermal stability in EMs. This theory has been used to evaluate the thermal stability of HMX, RDX, CL-20, CL-20/HMX and et al., and the theoretical analysis results proved to be in good agreement with the experimental tests [40,41].

#### 3.2.1. Trigger Bond Length

For the energetic material system with more components, the study of its thermal stability should focus on the most explosive components of the structural changes. According to the “minimum bond level principle”, the smaller the bond level of the trigger bond, the less thermally stable the system. A longer bond length corresponds to a smaller bond level of the trigger bond, thereby reducing the system’s thermal stability.

It is reported that the N–C bond between the five-membered ring and NO_2_ and the other is the N–O bond in the five-membered ring are the most unstable bonds in the DNTF molecule [42]. Therefore, changes in the thermal stability of the system can be inferred from variations in the maximum bond lengths of the N–C and N–O bonds.

The bond length distributions of the N–C bond and N–O in the DNTF (1 1 1) system and the DNTF (1 1 1)/F2603 composite system at different temperatures are given in Figures S1 and S2, respectively. From Figures S1 and S2, it can be seen that the N–C bond has the longest bond length by comparing the bond lengths of N–C and N–O in the two systems, so it is more likely that the N–C bond is the trigger bond.

**Figure 4:**
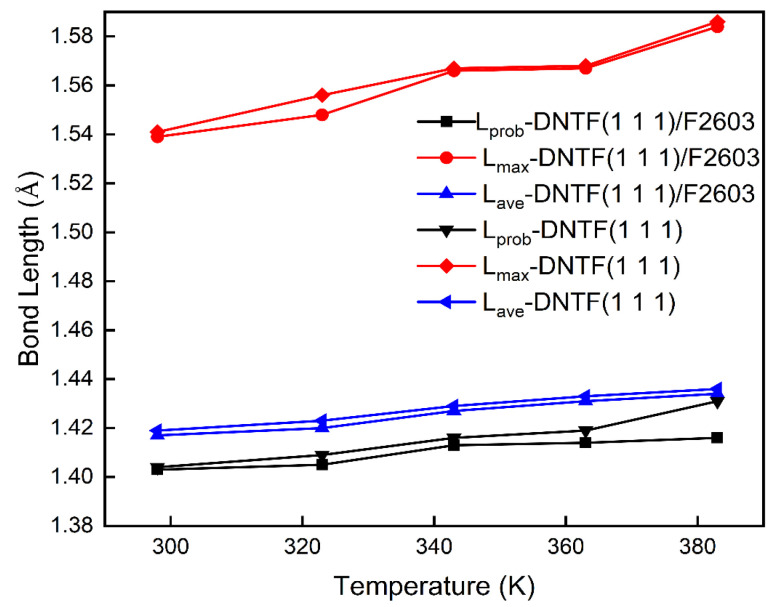
Variation in the trigger bond lengths of N–C bonds with temperature in the DNTF (1 1 1) system and the DNTF (1 1 1)/F2603 composite system.

The maximum bond length (*L*_max_), the average bond length (*L*_ave_), and the most probable bond length (*L*_prob_) of N–C and N–O bonds in the DNTF (1 1 1) system and DNTF (1 1 1)/F2603 composite system at different temperatures are given in Tables S1 and S2, respectively. Figure 4 shows the variation in *L*_max_, *L*_ave_, and *L*_prob_ with temperature in the DNTF (1 1 1) system and the DNTF (1 1 1)/F2603 composite system.

From Figure 4, it can be seen that the *L*_prob_, *L*_ave_, and *L*_max_ of the N–C bonds become longer with the increasing temperature within 298 K–398 K. This indicates that the thermal stability of both systems decreases as the temperature increases. It is consistent with the experimental fact that thermal stability decreases with increasing temperature. It can also be seen that although *L*_prob_, *L*_ave_, and *L*_max_ in both systems increase with temperature, *L*_max_ increases the most. This is because with the temperature increasing, the atoms vibrate more vigorously to deviate more easily from their equilibrium positions. Thus, the part of the molecule with the largest bond length is “activated”. Therefore, the *L*_max_ of the trigger bond can be used as one of the theoretical bases for judging the advantages and disadvantages of the thermal stability of the two systems. From the comparison in Figure 4, it can also be seen that the *L*_prob_, *L*_ave_, and *L*_max_ of the DNTF (1 1 1)/F2603 composite system are lower than those of the DNTF (1 1 1) system when the temperature is in the range of 298 K–398 K. The lower bond strength in the DNTF(111)/F2603 system can be attributed to the interactions between F2603, which has a high heat capacity, and the DNTF(111) molecules. In particular, the presence of F2603 may lead to the formation of new molecular bonds within the composite system. The interactions between these new bonds influence the bonding strength of DNTF(111), thereby affecting its thermal stability. This suggests that the presence of the polymeric passivator F2603 plays an important role in improving the thermal stability of DNTF.

#### 3.2.2. Cohesive Energy Density

The external energy required for a mole of a substance to remove all the forces between molecules is defined as cohesive energy. Cohesive energy density (CED) is the energy required by 1 mol of condensate per unit volume to overcome intermolecular vaporization, and it is a physical quantity to evaluate the intermolecular interforce [43,44]. It is described in Equation (1).

(1) $$\mathrm{CED}=\frac{H_{v}-\mathrm{RT}}{V_{m}}$$

In Equation (1), *H*_v_ is the molar heat of vaporization; RT is the expansion work performed by the vaporization; and *V*_m_ is the molar volume.

Both cohesion energy and CED can be used to measure the magnitude of intermolecular interaction forces. For small molecule compounds, the cohesion energy is approximately equal to the heat of sublimation or constant volume heat of evaporation of the substance, so its CED can be directly estimated from thermodynamic data. In atomic simulations, the increase in energy required to eliminate all interactions between molecules of 1 mol of matter is defined as the cohesion energy. Similarly, the cohesion energy per unit volume is the CED. The unit volume was defined as the instantaneous volume of the simulation box at each timestep, normalized by the total number of particles in the system.

**Figure 5:**
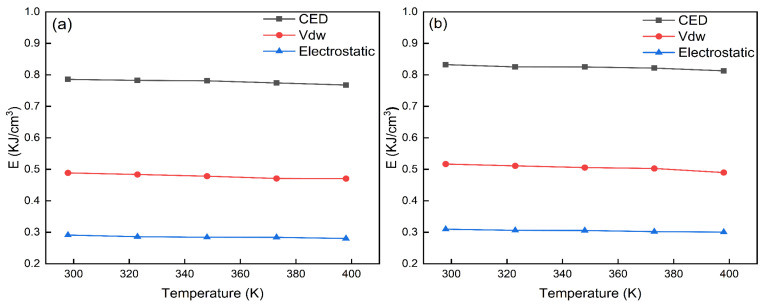
Variation in cohesive energy density and its component with temperature for DNTF (1 1 1) system (**a**) and DNTF (1 1 1)/F2603 composite system (**b**).

**Figure 6:**
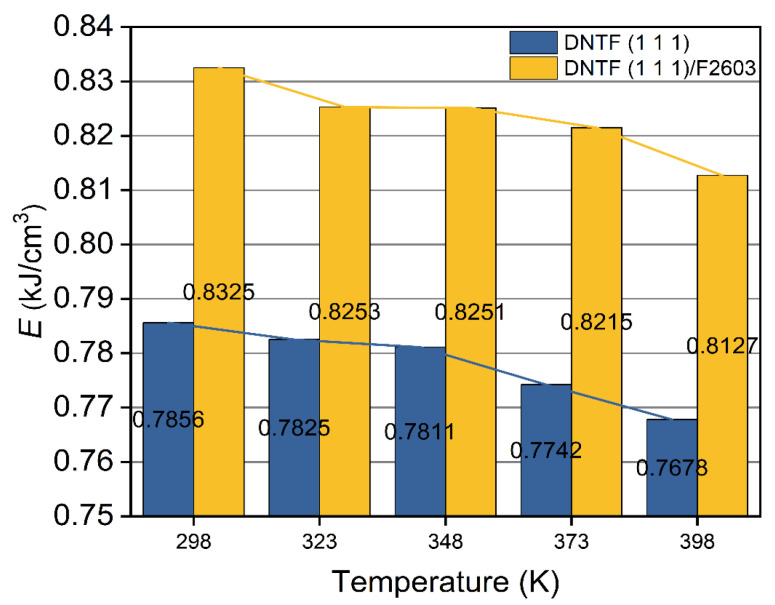
Comparison of cohesive energy density of DNTF (1 1 1) system and DNTF (1 1 1)/F2603 composite system.

The results of the cohesive energy density simulated for the DNTF (1 1 1) system and the DNTF (1 1 1)/F2603 composite system are shown in Tables S3 and S4. The variation in the CED and its components with temperature for the DNTF (1 1 1) system and the DNTF (1 1 1)/F2603 composite system are given in Figure 5. A comparative plot of the CED of the DNTF (1 1 1) system and the DNTF (1 1 1)/F2603 composite system is given in Figure 6.

From Tables S4 and S5 and Figure 5, the CED, vdW, and electrostatic force of the DNTF (1 1 1) and DNTF (1 1 1)/F2603 composite systems are monotonically decreasing with increasing temperature. This indicates that the energy required for these two systems to change from a crystalline state to a gaseous state is reduced and means that the thermal stability of these two systems become worse. This is consistent with the experimental fact that the thermal stability of the system gradually decreases with temperature. It can also be shown that CED can be used as one of the bases for the theoretical judgment of the thermal stability of the system. From Figure 6, it can also be seen that the CED of the composite system is larger than that of the pure matter system at the same temperature. The results show that the thermal stability of the mixed system is better than that of the pure substance system without F2603. This is consistent with the previous results obtained by trigger bond length simulations.

### 3.3. Thermal Properties

**Figure 7:**
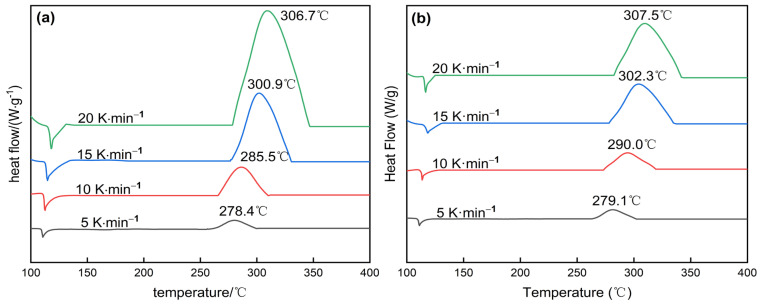
DSC curves of DNTF (**a**) and DNTF/F2603 (**b**) at four different heating rates.

Thermal properties, including thermal stability and thermal decomposition behavior, are key indicators of the performance of EMs and provide valuable insights for their practical applications [45]. In this study, the thermal decomposition of DNTF and DNTF/F2603 was investigated by differential scanning calorimetry (DSC) technique. Thus, the effect of F2603 on the thermal decomposition of DNTF was examined. Due to the high volatility of DNTF under ambient pressure, the thermal decomposition behavior could not be accurately assessed using aluminum crucibles with holes. Therefore, sealed crucibles were employed for the investigation of DNTF and DNTF/F2603 at heating rates of 5, 10, 15, and 20 K·min^−1^. The activation energies were calculated. The obtained DSC curves are shown in Figure 7. From Figure 7, the decomposition temperatures of DNTF and DNTF/F2603 increased with the increase in the heating rate. DNTF/F2603 and DNTF have similar thermal decomposition behaviors at the same heating rate. The addition of F2603 led to a slight increase in the exothermic peak temperature, although by less than 5 °C, indicating that F2603 is compatible with DNTF and contributes to enhancing its thermal stability.

To further investigate the effect of F2603 on the thermal decomposition behavior and thermal stability of DNTF, the kinetic parameters of the thermal decomposition reactions of the two samples were calculated by Kissinger’s Equation (2) and Ozawa’s Equation (3) at four different heating rates. The peak temperatures and the corresponding thermal kinetic data are summarized in Table 2.

(2) $$\ln\left(\frac{\beta }{T_{pi}^{2}}\right)=\ln\frac{AR}{E_{a}}-\frac{E_{a}}{RT_{pi}}$$

(3) $$\mathrm{lg}\beta_{i}+\frac{0.4567E_{o}}{RT}=C$$

In Equations (2) and (3), *β* is the heating rate; °C·min^−1^; *T*_pi_ is peak temperature, °C; *A* is the preexponential factor, s^−1^; *E* is the apparent activation energy, kJ·mol^−1^; and *R* is the gas constant; 8.314 J·(mol·K)^−1^.

From Table 2, the apparent activation energies of thermal decomposition of the two samples calculated by Kissinger formula and Ozawa formula are in good agreement, and the linear correlation coefficient is close to 0.99. It indicates that the calculation results are credible. Moreover, the *E*_a_ of DNTF/F2603 was 7.2 kJ·mol^−1^ higher than that of DNTF, indicating that a greater amount of energy is required for the decomposition of the composite. It indicates that the polymeric passivator F2603 retarded the thermal decomposition of DNTF and improved the thermal stability of DNTF under heating conditions. This result is consistent with the simulation analysis.

### 3.4. Mechanical Properties

Mechanical properties are one of the most important properties of EMs and are related to their preparation, processing, and use. The main parameters of mechanical properties include modulus of elasticity, coefficient of elasticity, and Poisson’s ratio. Tensile modulus (*E*), shear modulus (*G*), and bulk modulus (*K*) are the three engineering moduli of a material under different stresses [46]. *E* reflects the material’s rigidity and, within the elastic deformation range, represents the ratio of tensile force to strain. Generally, a higher *E* indicates greater material rigidity. *G* measures the material’s capacity for plastic deformation and is defined as the ratio of shear stress to shear strain within the elastic range. A smaller *G* value usually reflects the better plastic deformation capacity of a material. *K* indicates the material’s fracture behavior under external forces, representing the ratio of change in system pressure to the change in relative volume. The larger the K value is, the greater the fracture strength of the material will be; *K/G* indicates the ratio of the bulk modulus to the shear modulus of the system. The plastic deformation capacity associated with toughness can be measured by the magnitude of *K/G*, which is the ability of a material to withstand large deformations under impact loading or vibration loading without being damaged. The larger *K/G* value implies better plasticity and toughness of a material. Cauchy pressure (*C*_12_–*C*_44_) reflects the ductility of the material, as observed in its fracture surface morphology. When the value is negative, the material is poorly ductile and brittle. Meanwhile, the material is adequately ductile with a positive value. Poisson’s ratio (*γ*), defined as the negative ratio of transverse strain to longitudinal strain during material stretching, also gauges plasticity. *γ* values in 0.2~0.4 within the material are certainly plastic, conducive to processing. The above mechanical property parameters were all calculated using the Forcite module in Materials Studio software.

For smaller deformations in elastomers, the relationship between material stress and strain can be described using the generalized Hooke’s law [47], as follows:

(4) $$\sigma_{ij}=C_{ij}\varepsilon_{j},i,j=1,\cdots \cdots ,6$$

In Equation (4), $\sigma_{ij}$ is the stress tensor (GPa); $\varepsilon_{j}$ is the strain tensor (GPa); and $C_{ij}$ is the 6 × 6 elastic coefficient matrix. By idealizing the material as an isotropic medium, the elastic coefficient matrix describing the stress–strain behavior can be reduced to a matrix with only two independent variables (the Lamé coefficient) as shown in Equation (5).

(5) $$\left[\begin{array}{cccccc} \lambda +2\mu & \lambda & \lambda & 0 & 0 & 0\\ \lambda & \lambda +2\mu & \lambda & 0 & 0 & 0\\ \lambda & \lambda & \lambda +2\mu & 0 & 0 & 0\\ 0 & 0 & 0 & \mu & 0 & 0\\ 0 & 0 & 0 & 0 & \mu & 0\\ 0 & 0 & 0 & 0 & 0 & \mu \end{array}\right]$$

In Equation (5), λ and μ are the Lamé coefficients. Common mechanical parameters can be expressed by the coefficient of Lamé as Equation (6).

(6) $$\left\{\begin{array}{c} E=\mu \left(\frac{3\lambda +2\mu }{\lambda +\mu }\right)\\ K=\lambda +\frac{2}{3}\mu \\ G=\mu \\ \gamma =\frac{\lambda }{2\left(\lambda +\mu \right)} \end{array}\right.$$

The relationship between Poisson’s ratio γ and different moduli can be expressed as Equation (7).

(7) $$E=2G\left(1+\gamma \right)=3K\left(1-2\gamma \right)$$

**Figure 8:**
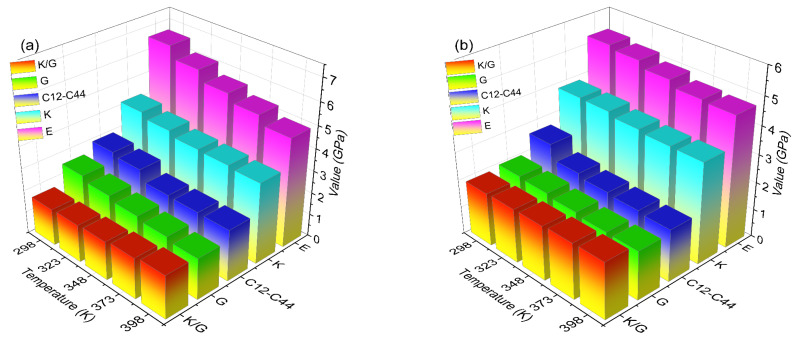
The variation in mechanical properties with temperature for the DNTF (1 1 1) (**a**) and DNTF (1 1 1)/F2603 (**b**) systems.

The specific mechanical property parameters of DNTF (1 1 1) system and the DNTF (1 1 1)/F2603 composite system are shown in Table S5 and Table S6, respectively, based on the above method. The mechanical properties of the DNTF (1 1 1) system and the DNTF (1 1 1)/F2603 composite system at different temperatures are given in Table 3 and Table 4, respectively. The variation in the mechanical properties with a temperature for the DNTF (1 1 1) system and the DNTF (1 1 1)/F2603 composite system are given in Figure 8.

From Table 3 and Table 4 and Figure 8, it can be seen that the values of *E*, *K*, and G for the DNTF (1 1 1) system and the DNTF (1 1 1)/F2603 composite system gradually decrease with increasing temperature in the range of 298 K to 398 K. This trend suggests a reduction in rigidity and hardness, accompanied by an increase in plasticity for both systems. These findings are consistent with the expected variation in mechanical properties with temperature. The positive values of *C*_12_–*C*_44_ for both systems within this temperature range indicate good ductility. Additionally, the *K*/*G* ratio for both systems increases with temperature, implying enhanced toughness. The *γ* value for both systems falls between 0.2 and 0.4, indicating moderate plasticity, which facilitates processing.

**Figure 9:**
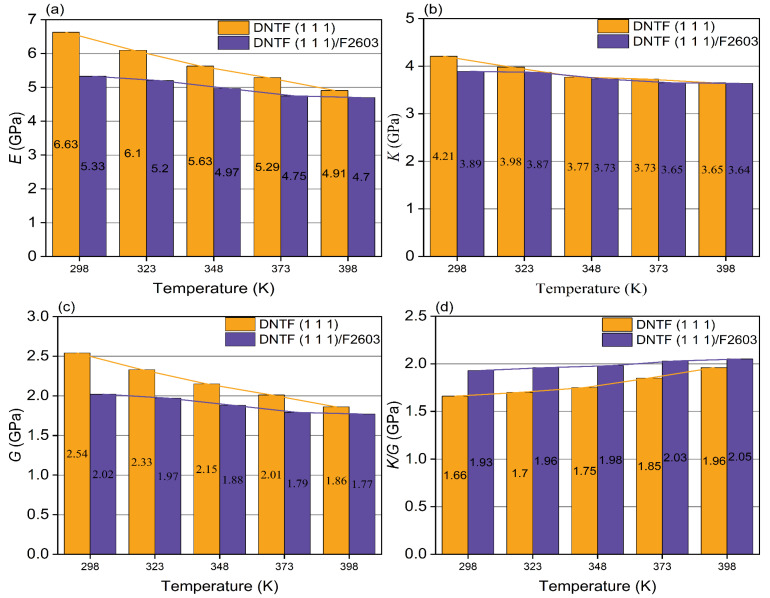
Comparison of (**a**) *E*, (**b**) *K*, (**c**) *G*, (**d**) *K*/*G* of DNTF (1 1 1) system and DNTF (1 1 1)/F2603 composite system.

Figure 9 illustrates the mechanical properties of the two studied systems. Figure 9a–c shows that the *E*, *K*, and *G* values of the DNTF (1 1 1)/F2603 composite system are lower than those of the DNTF (1 1 1) system across the temperature range of 298 K to 398 K. Thus, F2603 is capable of reducing the hardness and brittleness of DNTF (1 1 1) system and increasing the plasticity of DNTF (1 1 1). In addition, compared with the DNTF (1 1 1)/F2603 system, the values of *E*, *K*, and *G* for the DNTF (1 1 1) system decreased significantly with temperature. It shows that the resistance of the material to deformation is greatly influenced by temperature and the resistance of elastic deformation is weakened. The values of *E*, *K*, and *G* for the DNTF (1 1 1)/F2603 composite system tend to decrease little with temperature, suggesting that the addition of F2603 strengthens the material’s resistance to elastic deformation. Figure 9d is the comparison of the *K/G* of the DNTF (1 1 1) system and the DNTF (1 1 1)/F2603 composite system. The *K/G* of the DNTF (1 1 1)/F2603 composite system is greater than that of the DNTF (1 1 1) system, indicating that the addition of F2603 enhances the ductility of the DNTF (1 1 1) system.

## 4. Conclusions

Molecular dynamics simulations were carried out for the DNTF (1 1 1) system and the DNTF (1 1 1)/F2603 composite system to investigate the effect of temperature on the thermal stability and mechanical properties of the systems. The thermal stability of the two systems were also investigated experimentally using the DSC technique. The conclusions are as follows:

(1) Both theoretical and experimental studies indicated that the addition of F2603 makes the most probable bond length (*L_prob_*), the average bond length (*L_ave_*), and the maximum bond length (*L_max_*) of the DNTF (1 1 1) system become shorter and the CED become larger. Meantime, both the exothermic decomposition peak temperature and the activation energy of DNTF increased. Therefore, polymeric passivator F2603 contributes significantly to improving the thermal stability of DNTF.
(2) As the temperature increases, the modulus of both systems decreases, which means the hardness and the brittleness decreases, while the elasticity and the plasticity increase. The DNTF (1 1 1)/F2603 composite system was found to have better ductility, toughness, and impact resistance than the DNTF (1 1 1) system based on the mechanical properties’ calculations, which indicates that F2603 can greatly improve the mechanical properties of DNTF (1 1 1).

## Figures and Tables

**Table 1:** The comparison of the experimental and optimized lattice parameters of DNTF.

Lattice	a (Å)	b (Å)	c (Å)	α (°)	*β* (°)	*γ* (°)	*ρ* (g·cm^−3^)
Exp	6.662	10.740	15.093	90	90	90	1.937
COMPASS	6.632	10.965	14.962	90.00	90.00	90.00	1.870
Relative error/%	−0.45	2.1	−0.87	0	0	0	−3.5

**Table 2:** The thermal decomposition kinetic parameters of DNTF and DNTF/F2603.

Samples	*β* ^a^ (°C·min^−1^)	*T*_pi_ ^b^ (°C)	*E*_k_ ^c^ (kJ·mol^−1^)	logA_k_ ^d^ (s^−1^)	r_k_ ^e^	*E*_o_ ^f^ (kJ·mol^−1^)	r_o_ ^g^	*E*_a_ ^h^ (kJ·mol^−1^)
DNTF	5	278.4	107.9	7.83	0.96	111.6	0.97	109.8
	10	285.5						
	15	300.9						
	20	306.7						
DNTF/F2603	5	279.1	115.3	8.51	0.99	118.6	0.99	117.0
	10	290.0						
	15	302.3						
	20	307.5						

^a^ Heating rate. ^b^ Peak temperature. ^c^ Activation energy calculated by Kissinger method using peak temperature. ^d^ Pre-exponential factor. ^f^ Activation energy calculated by Ozawa method using peak temperature. ^h^ *E_a_* is the average of *E_k_* and *E_o_*. ^e,g^ Linear correlation coefficient.

**Table 3:** Mechanical properties of DNTF (1 1 1) systems at different temperatures.

T (K)	*G* (GPa)	*K/G*	*C*_12_–*C*_44_ (GPa)	*K* (GPa)	*E*(GPa)	*γ*
298	2.54	1.66	2.97	4.21	6.63	0.30
323	2.33	1.70	2.84	3.98	6.10	0.31
348	2.15	1.75	2.37	3.77	5.63	0.31
373	2.01	1.85	2.34	3.73	5.29	0.31
398	1.86	1.96	2.32	3.65	4.91	0.32

**Table 4:** Mechanical properties of the DNTF (1 1 1)/F2603 composite system at different temperatures.

T (K)	*G* (GPa)	*K/G*	*C*_12_–*C*_44_ (GPa)	*K* (GPa)	*E* (GPa)	*γ*
298	2.02	1.93	2.68	3.89	5.33	0.32
323	1.97	1.96	2.15	3.87	5.20	0.32
348	1.88	1.98	2.02	3.73	4.97	0.32
373	1.79	2.03	1.96	3.65	4.75	0.32
398	1.77	2.05	1.89	3.64	4.70	0.32

## Data Availability

Data are contained within the article.

## Supplementary Materials

The following supporting information can be downloaded at: https://www.mdpi.com/article/10.3390/molecules30081665/s1, Figure S1: Bond length distributions of N–C and N–O in DNTF (1 1 1) system at 298 K (a), 323K (b), 348 K (c), 373 K (d) and 398 K (e); Figure S2: Bond length distributions of N–C and N–O in DNTF (1 1 1)/F2603 composite system at 298 K (a), 323K (b), 348 K (c), 373 K (d) and 398 K (e); Table S1: Trigger bond lengths of equilibrium structures in DNTF (1 1 1) system at different temperatures; Table S2: Trigger bond lengths of explosive components in equilibrium structure of DNTF (1 1 1)/F2603 composite system at different temperatures; Table S3: Cohesive energy density and its components for DNTF (1 1 1) system at different temperatures; Table S4: Cohesive energy density and its components for DNTF (1 1 1)/F2603 composite system at different temperatures; Table S5: Mechanical properties of DNTF (1 1 1) systems at different temperatures; Table S6: Mechanical properties of DNTF (1 1 1)/F2603 composite system at different temperatures.
