# Supplementary material for: Polymeric Desensitizer Fluororubber: A Good Binder to Improve the Thermal Stability and Mechanical Properties of 3,4-Dinitrofurazanfuroxan
Source: Molecules. 2025 Apr 8;30(8):1665. doi: 10.3390/molecules30081665 (PMC12029978; doi:10.3390/molecules30081665)
Supplement: Supplementary file 1 [file molecules-30-01665-s001.zip › molecules-3546044-supplementary.pdf]

Supporting Information

Polymeric Desensitizer Fluororubber: A Good Binder to Improve the Thermal Stability and Mechanical Properties of 3,4-Dinitrofurazanfuroxan

Shenghui Wang [1,†], Xiaogang Mu [1,*,†], Yiming Luo [2], Ronghui Ju [2], Xuanjun Wang [1,*], Haixia Ma [3] and Jijun Xiao [4]

[1] Zhijian Laboratory, Rocket Force University of Engineering, Xi'an 710025, China; wshalano@163.com
[2] Xi'an Modern Chemistry Research Institute, Xi'an 710065, China; iamrlym@126.com (Y.L.); ronghuiju@126.com (R.J.)
[3] Xi'an Key Laboratory of Special Energetic Materials, School of Chemical Engineering, Northwest
University, Xi'an 710069, China; mahx@nwu.edu.cn
[4] Molecules and Materials Computation Institute, School of Chemical Engineering, Nanjing University of Science and Technology, Nanjing 210094, China
* Correspondence: muxg2001@163.com (X.M.); wangxj503@sina.com (X.W.)
† These authors contributed equally to the work.

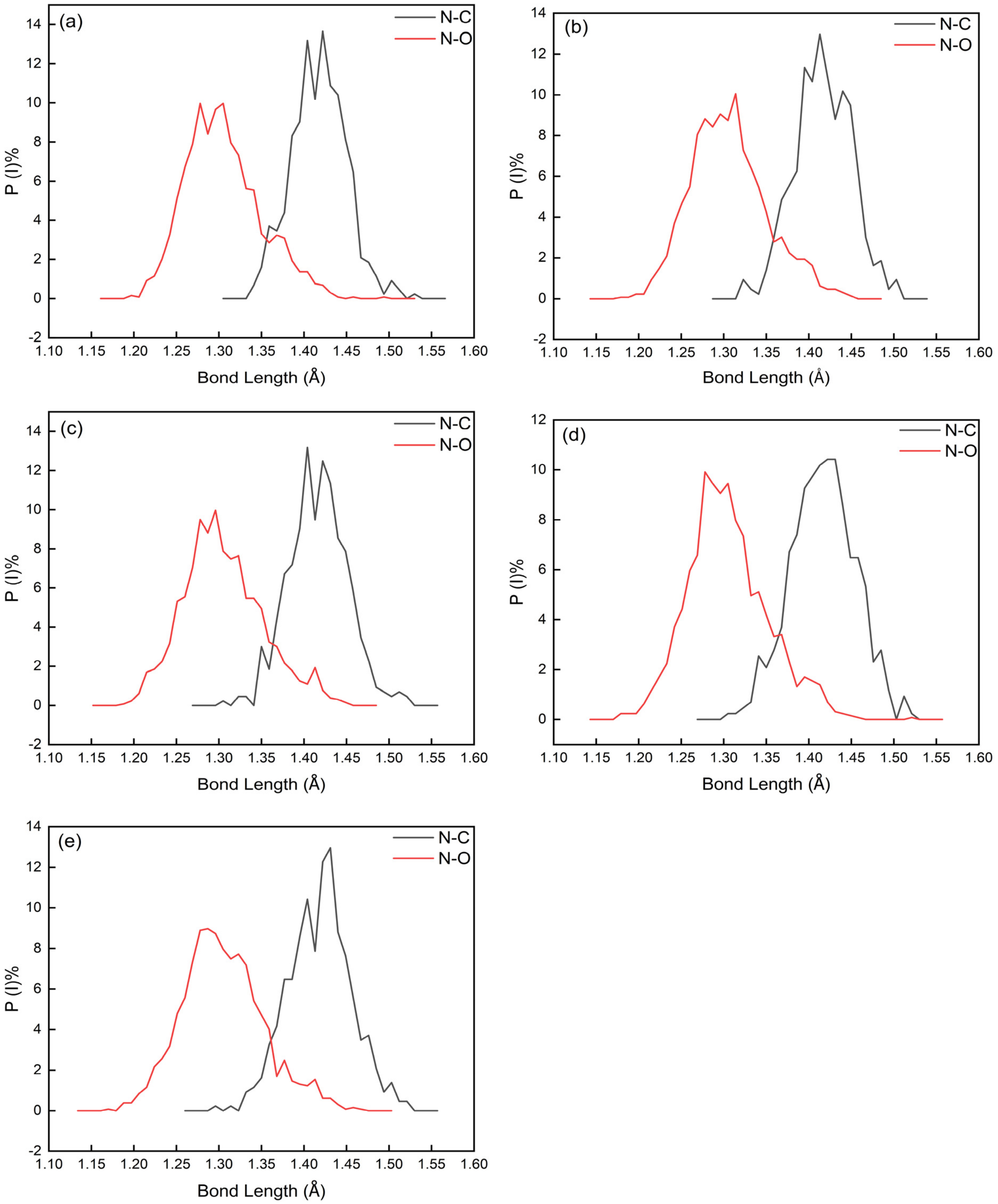

Figure S1. Bond length distributions of N–C and N–O in DNTF (1 1 1) system at 298 K (a), 323K (b), 348 K (c), 373 K (d) and 398 K (e)

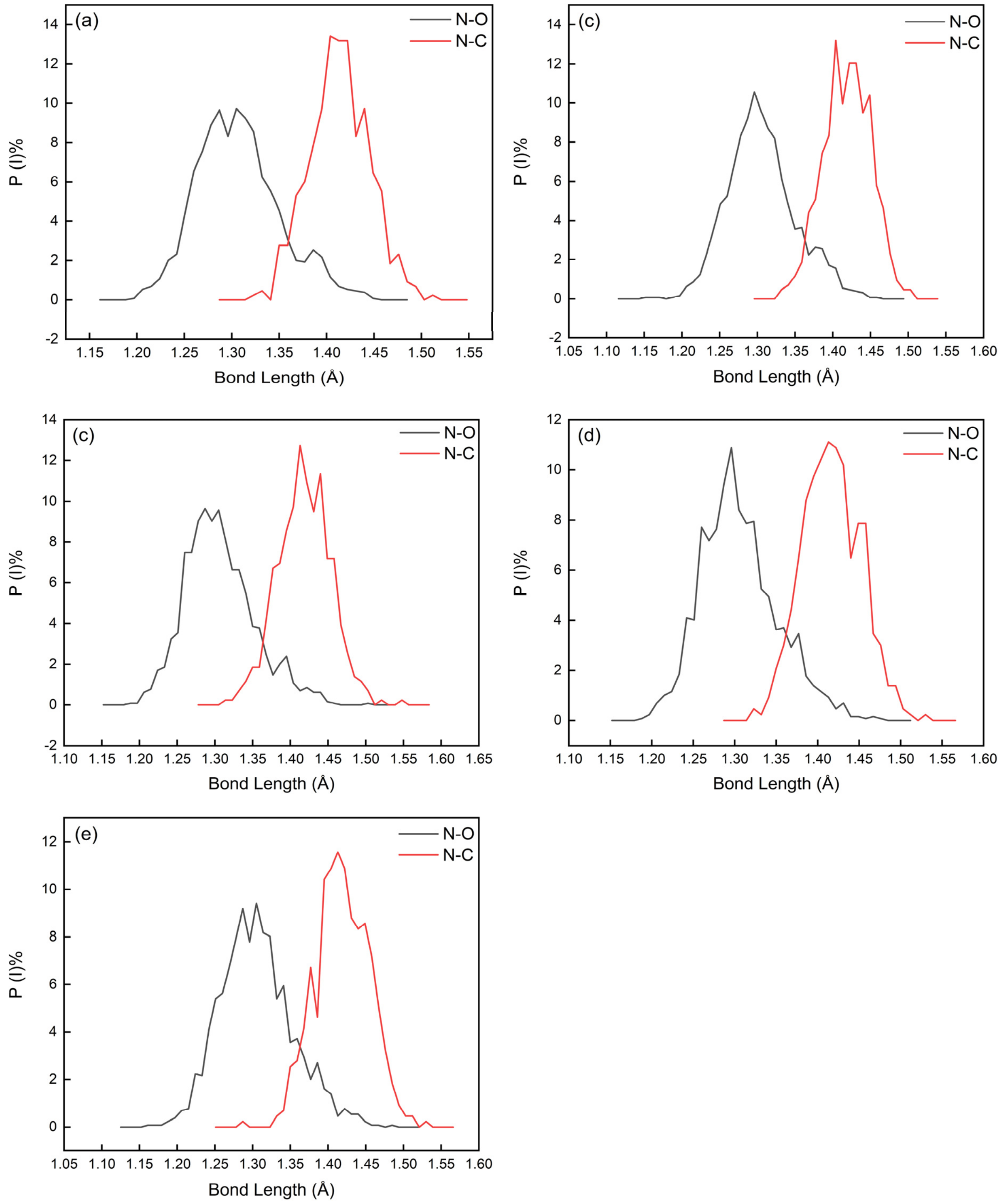

Figure S2. Bond length distributions of N–C and N–O in DNTF (1 1 1)/F2603 composite system at 298 K (a), 323K (b), 348 K (c), 373 K (d) and 398 K (e)

Table S1. Trigger bond lengths of equilibrium structures in DNTF (1 1 1) system at different temperatures

T (K)	Bong length (Å)	N–O	N–C
	L_{prob}	1.288	1.404
298	L_{max}	1.489	1.541
	L_{ave}	1.314	1.419
	L_{prob}	1.299	1.409
323	L_{max}	1.495	1.556
	L_{ave}	1.329	1.423
	L_{prob}	1.306	1.416
348	L_{max}	1.513	1.567
	L_{ave}	1.329	1.429
	L_{prob}	1.310	1.419
373	L_{max}	1.530	1.568
	L_{ave}	1.345	1.433
	L_{prob}	1.314	1.431
398	L_{max}	1.557	1.586
	L_{ave}	1.350	1.436

Table S2. Trigger bond lengths of explosive components in equilibrium structure of DNTF (1 1 1)/F2603 composite system at different temperatures

T (K)	Bond Length (Å)	N–O	N–C
	L_{prob}	1.287	1.403
298	L_{max}	1.485	1.539
	L_{ave}	1.305	1.417
	L_{prob}	1.296	1.405
323	L_{max}	1.494	1.548
	L_{ave}	1.323	1.420
	L_{prob}	1.305	1.413
348	L_{max}	1.512	1.566
	L_{ave}	1.326	1.427
	L_{prob}	1.306	1.414
373	L_{max}	1.521	1.567
	L_{ave}	1.332	1.431
	L_{prob}	1.309	1.416
398	L_{max}	1.539	1.584
	L_{ave}	1.346	1.434

Table S3. Cohesive energy density and its components for DNTF (1 1 1) system at different temperatures

T (K)	298	323	348	373	398

CED ($kJ \cdot cm^{-3}$)	0.7856	0.7825	0.7811	0.7742	0.7678
vdW ($kJ \cdot cm^{-3}$)	0.4887	0.4837	0.4781	0.4710	0.4706
Electrostatic ($kJ \cdot cm^{-3}$)	0.2912	0.2860	0.2843	0.2840	0.2803

Table S4. Cohesive energy density and its components for DNTF (1 1 1)/F2603 composite system at different temperatures

T (K)	298	323	348	373	398
CED ($kJ \cdot cm^{-3}$)	0.8325	0.8253	0.8251	0.8215	0.8127
vdW ($kJ \cdot cm^{-3}$)	0.5168	0.5111	0.5056	0.5026	0.4898
Electrostatic ($kJ \cdot cm^{-3}$)	0.3099	0.3062	0.3057	0.3021	0.3007

Table S5. Mechanical properties of DNTF (1 1 1) systems at different temperatures

T (K)	298	323	348	373	398
C_{11}	8.83	7.40	6.98	6.97	6.90
C_{22}	8.96	8.75	8.18	7.46	6.54
C_{33}	9.32	8.92	8.15	7.83	7.60
C_{44}	2.43	2.21	1.99	1.93	1.81
C_{55}	2.21	1.98	1.96	1.89	1.76
C_{66}	2.99	2.81	2.51	2.22	2.01
C_{12}	5.40	5.05	4.36	4.27	4.13
C_{13}	4.69	4.41	3.87	3.79	3.64
C_{23}	4.46	4.20	3.90	3.69	3.50
C_{12}-C_{44}	2.97	2.84	2.37	2.34	2.32
λ	3.95	3.69	3.46	3.40	3.29
μ	2.54	2.33	2.15	2.01	1.86

Table S6. Mechanical properties of DNTF (1 1 1)/F2603 composite system at different temperatures

T (K)	298	323	348	373	398
C_{11}	7.15	6.97	6.95	6.93	6.92
C_{22}	7.92	7.81	6.95	6.69	6.62
C_{33}	7.73	7.62	7.50	6.96	6.89
C_{44}	1.87	1.86	1.76	1.75	1.73
C_{55}	1.85	1.80	1.68	1.65	1.63
C_{66}	2.34	2.25	2.20	1.98	1.96
C_{12}	4.55	4.01	3.78	3.67	3.57
C_{13}	4.34	3.78	3.45	3.23	3.19
C_{23}	3.80	3.72	3.45	3.22	3.18
C_{12}-C_{44}	2.68	2.15	2.02	1.96	2.89
λ	3.56	3.53	3.37	3.27	3.26
μ	2.02	1.97	1.88	1.79	1.77
